# Supplementary material for: Biomacromolecules enabled dendrite-free lithium metal battery and its origin revealed by cryo-electron microscopy
Source: Nat Commun. 2020 Jan 24;11:488. doi: 10.1038/s41467-020-14358-1 (PMC6981142; doi:10.1038/s41467-020-14358-1)
Supplement: Supplementary file 1 — Supplementary Information [file 41467_2020_14358_MOESM1_ESM.doc]

**Supporting information**

**Biomacromolecules enabled dendrite-free lithium metal battery and its origin revealed by cryo-electron microscopy**

Ju et al

**Supplementary Figures**


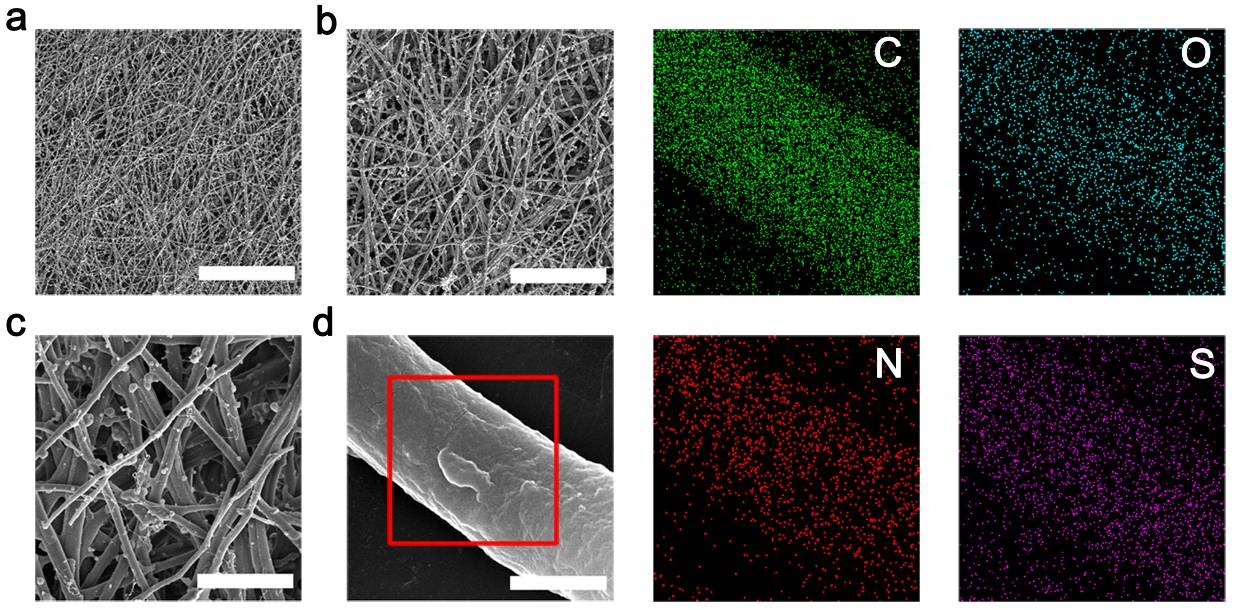


**Supplementary Figure 1.** Characterization of eggshell membrane from the top view. **a-c** SEM images of eggshell membrane (ESM) with various magnifications. **d** Scanning electron microscopy (SEM) image of single fiber in ESM the corresponding elemental mapping of C, O, N, and S. Scale bars, (**a**) 100 μm, (**b**) 50 μm, (**c**) 10 μm, and (**d**) 1 μm.


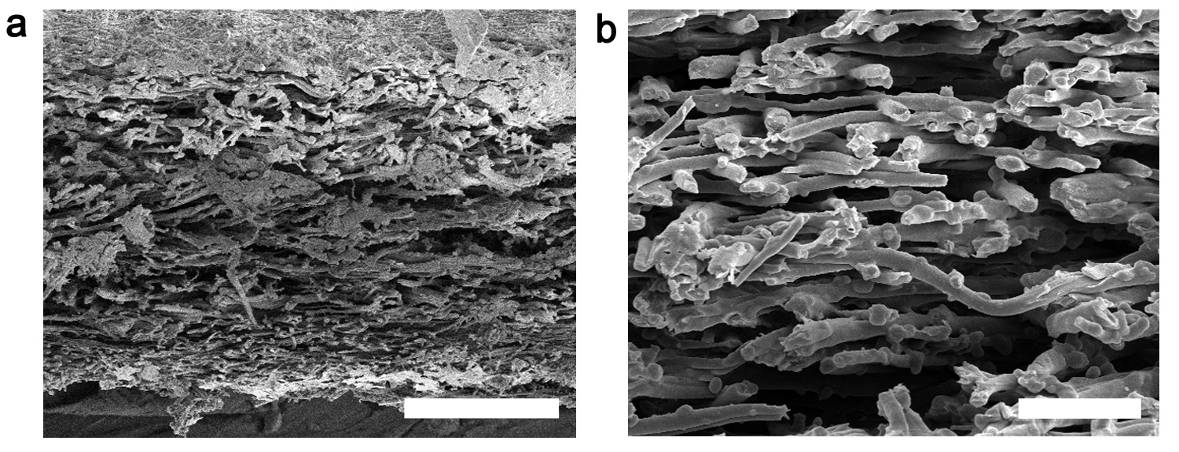


**Supplementary Figure 2.** Characterization of eggshell membrane from the cross-section view. SEM images of (**a**) cross-sectional ESM and (**b**) its corresponding enlarged area. Scale bars, (**a**) 50 μm, and (**b**) 10 μm.


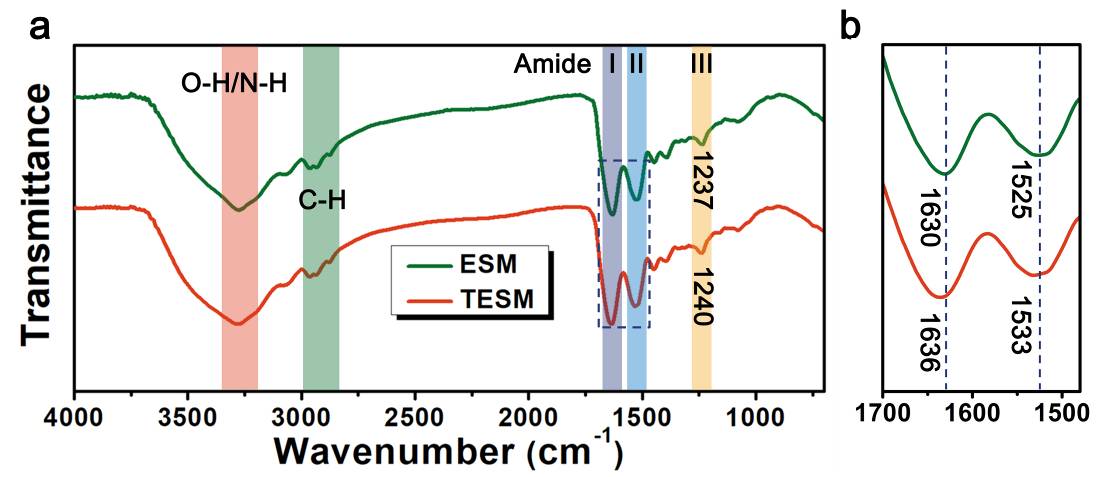


**Supplementary Figure 3.** [Structural](javascript:;) [transformation](javascript:;) of ESM after modification. **a** Fourier transformation infrared spectroscopy (FTIR) spectra of the pristine ESM and trifluoroethanol modified eggshell membrane (TESM). **b** Detailed information of FTIR amide I and II spectra of ESM and TESM. Typical protein absorption bands are exhibited at 1630, 1525, and 1237 cm^−1^ in FTIR spectrum of the pristine ESM, assigned as amide I (C=O stretching), amide II (N-H in plane bending/C-N stretching) and amide III absorbance, respectively. They present the characteristics of β-sheet type structures.^1,2^ In addition, the other four peaks can be observed: 3275 cm^−1^ (O-H and N-H stretching), 2930 cm^−1^ (C-H stretching), 1393 cm^−1^ (COO^-^ symmetric stretch) and 1449 cm^−1^ (CH_2_ scissoring).^3,4^ Peaks of amide groups shift after the solvothermal treatment in TFEA solution, whereas other peaks basically remain unchanged.


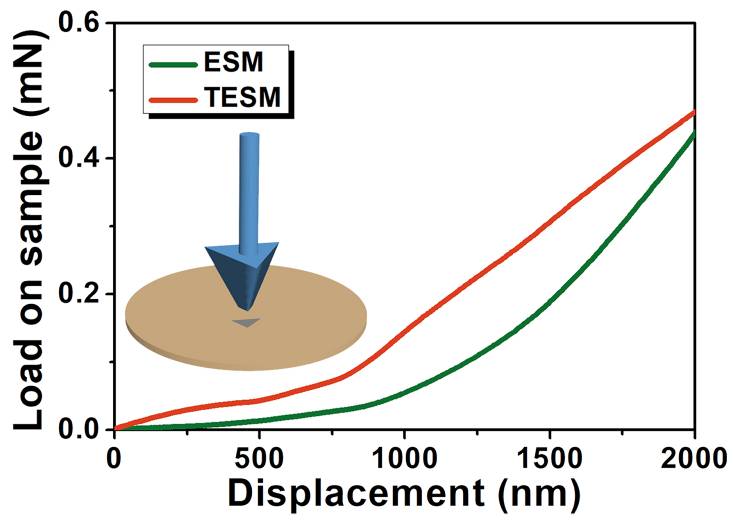


**Supplementary Figure 4.** Load–displacement curves of ESM and TESM during loading process measured by nanoindentation test.


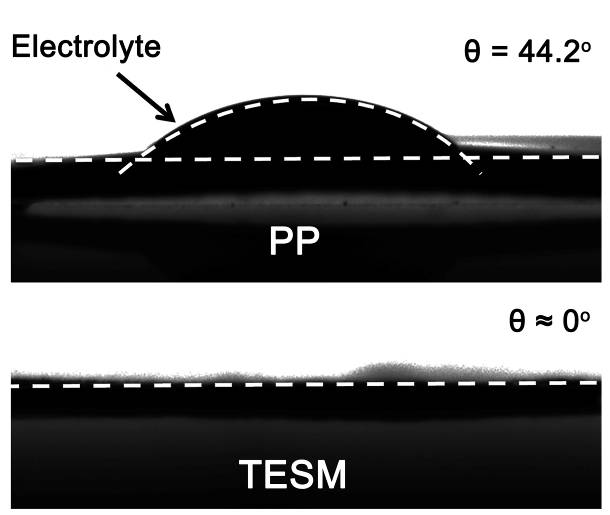


**Supplementary Figure 5.** Contact angles between ether-based electrolyte and polypropylene (PP) separator or TESM layer.


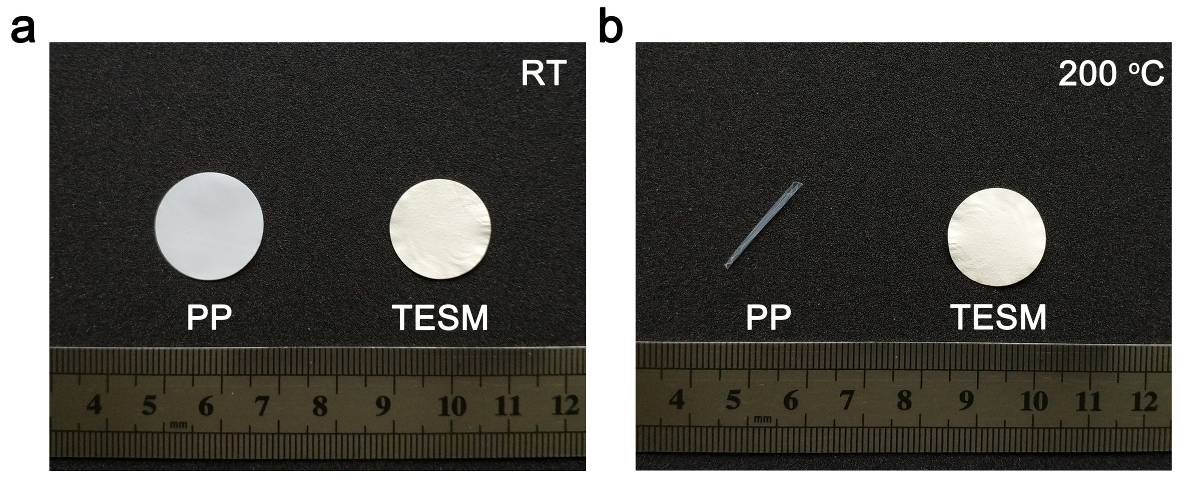


**Supplementary Figure 6.** Heat deterioration test of TESM. Digital Photographs of heat deterioration test of PP separator and TESM layer at (**a**) room temperature and (**b**) 200 ^o^C for 1 h.


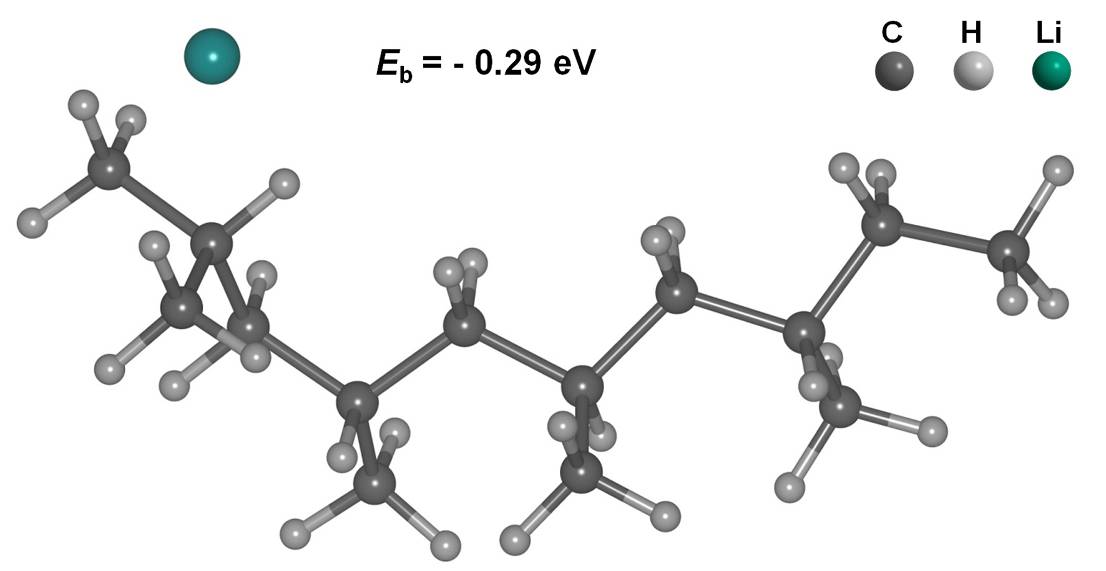


**Supplementary Figure 7.** The optimized geometry of Li binding on PP separator. The negative binding energy of – 0.29 eV refers to a poor lithium affinity of PP.


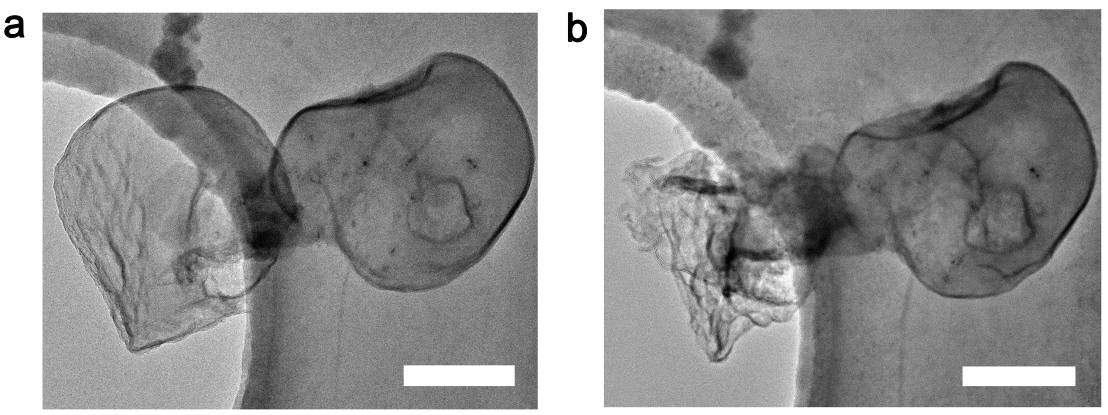


**Supplementary Figure 8.** Standard TEM images of Li deposits. Transmission electron microscopy (TEM) images of Li nuclei (**a**) before and (**b**) after attempting constant electron-beam irradiation in normal TEM at room temperature. Scale bars, (**a, b**) 500 nm.


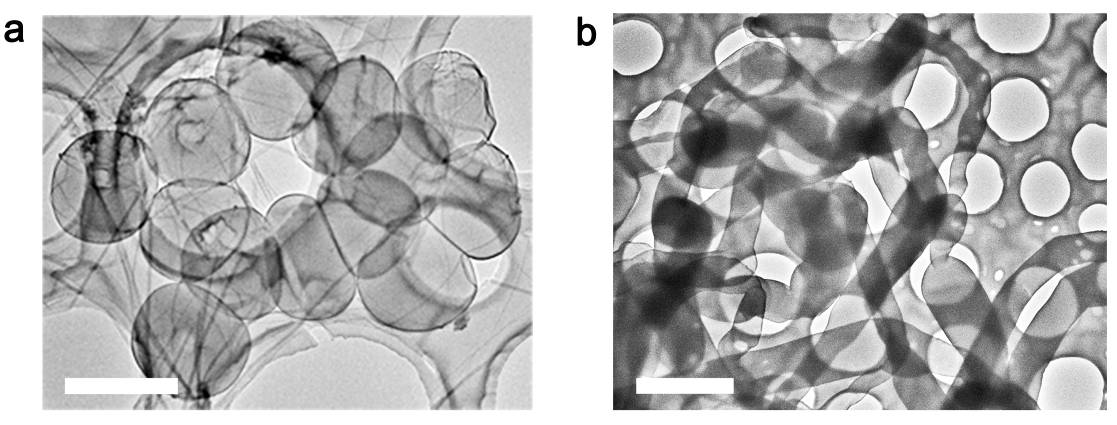


**Supplementary Figure 9.** Cryo-TEM images of Li deposites. Cryo-TEM images of (**a**) Li microspheres formed assisted by TESM and (**b)** Li dendrites on TESM-free Cu grid after the first deposition in ether-based electrolytes. Scale bars, (**a**) 2 μm, and (**b**) 5 μm.


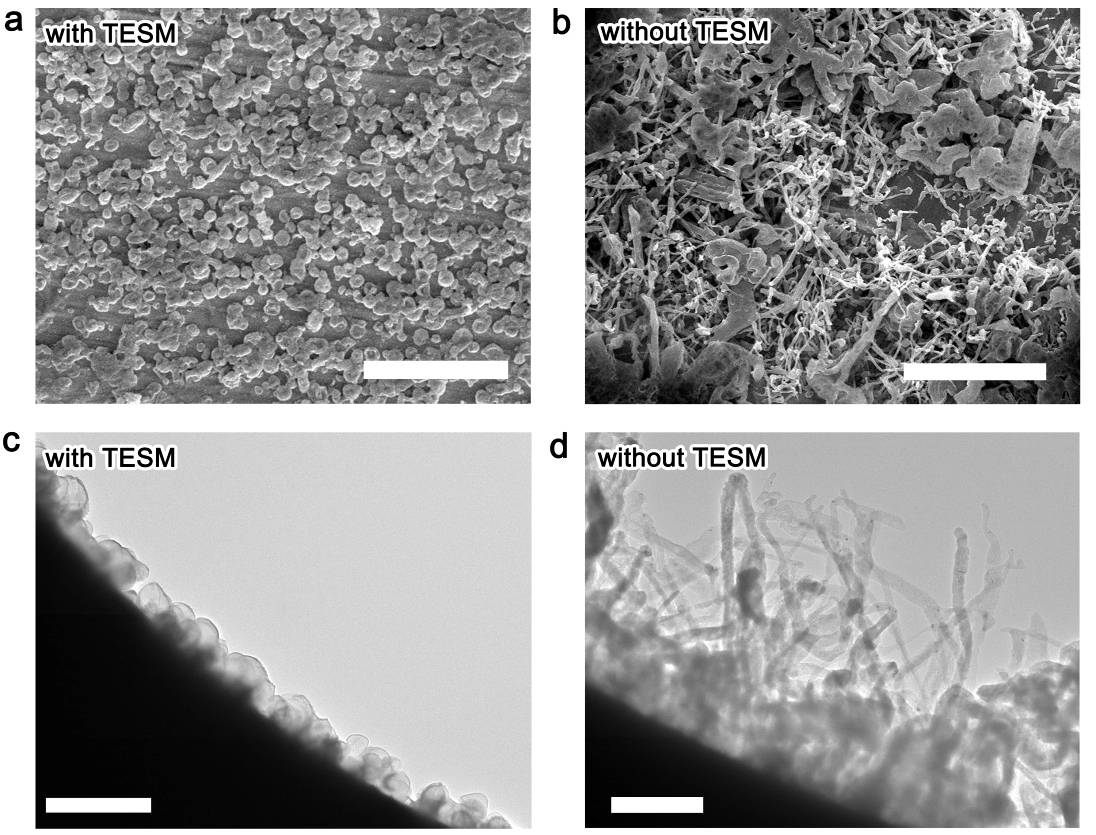


**Supplementary Figure 10.** Li deposits assisted by TESM in carbonate-based electrolytes. SEM and Cryo-TEM images of Li deposits **(a, c)** with and **(b, d)** without TESM at a current density of 0.5 mA cm^−2^ with a capacity of 0.1 mAh cm^−2^ in carbonate-based electrolytes. Scale bars, (**a, b**) 10 μm, (**c**) 5 μm and (**d**) 2 μm.


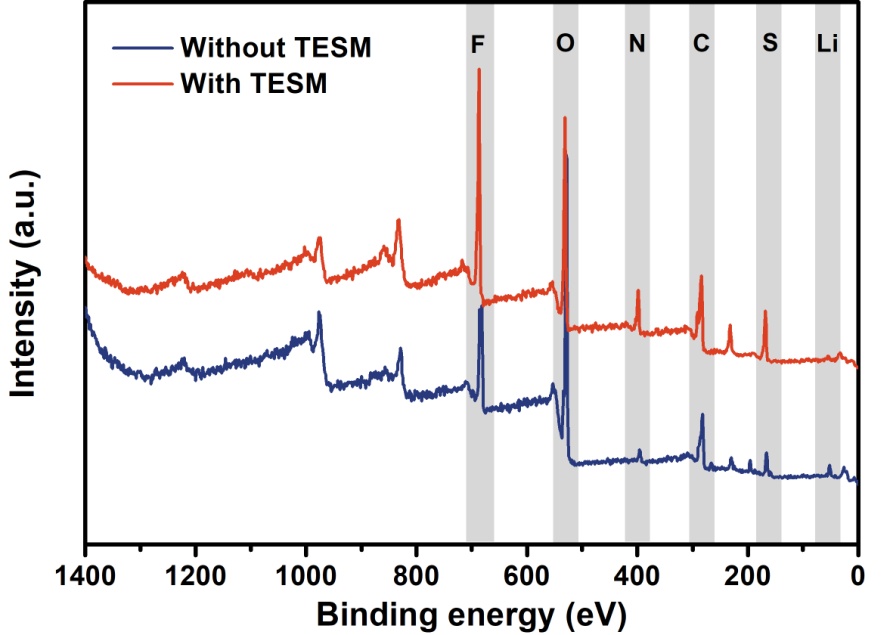


**Supplementary Figure 11.** XPS characterizations of Li foil coated without and with TESM after 20 cycles at 2 mA cm^−2^ with a capacity of 1 mAh cm^−2^ in ether-based electrolytes.


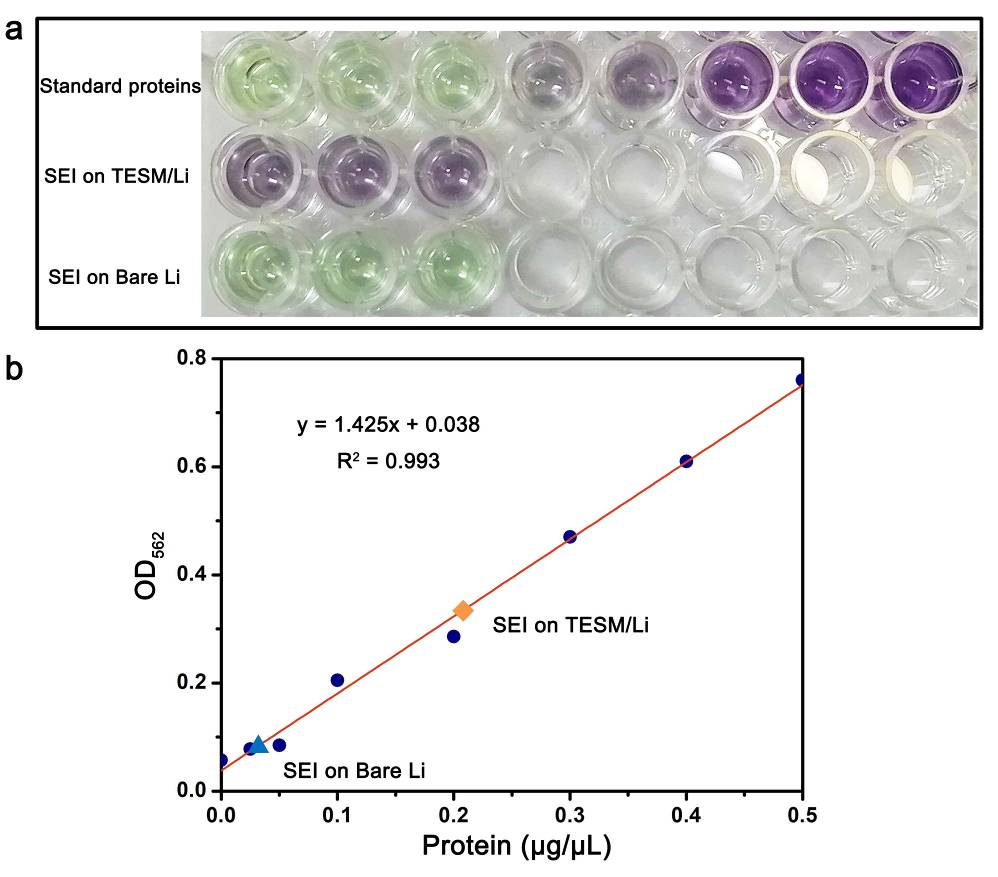


**Supplementary Figure 12.** BCA protein assay for SEI on TESM/Li. **a** The BCA assay showing the typical chromogenic reaction of standard proteins, SEI on TESM/Li, and SEI on bare Li in BCA reagent. The color of standard sample changes from pale green to deep purple as the protein concentration increases. **b** The curve of the absorbance at 562 nm vs protein concentration is plotted by the standard protein sample in (**a**), where the protein concentrations of the SEI on TESM/Li or bare Li are highlighted (The protein concentration of SEI on TESM/Li is 0.207 μg/μL and the SEI on bare Li did not contain protein within the error range).


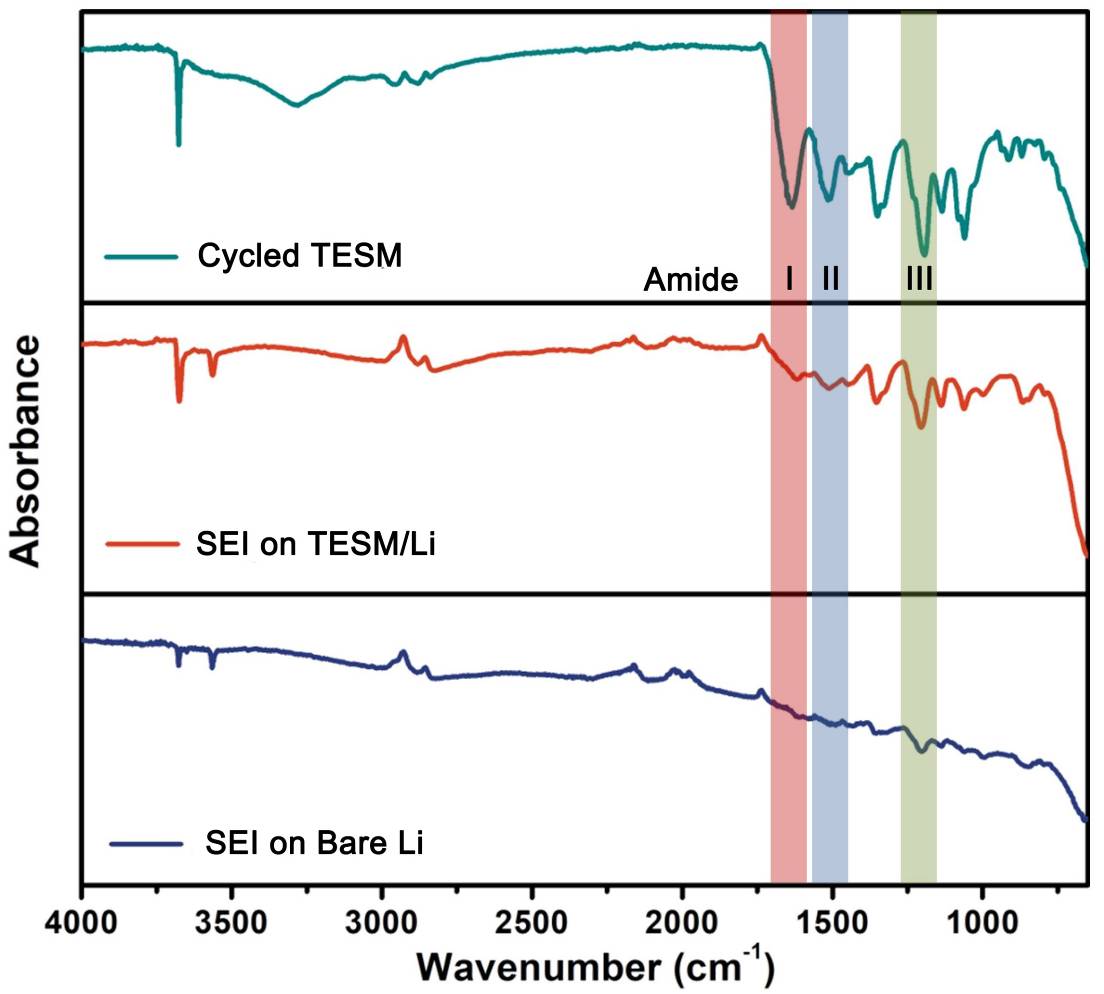


**Supplementary Figure 13.** FTIR spectra of the TESM, SEI on TESM/Li, and SEI on bare Li after 20 cycles at 2 mA cm^−2^ with 1 mAh cm^−2^ in ether-based electrolytes, where the typical bands of amide I, II, III are highlighted.


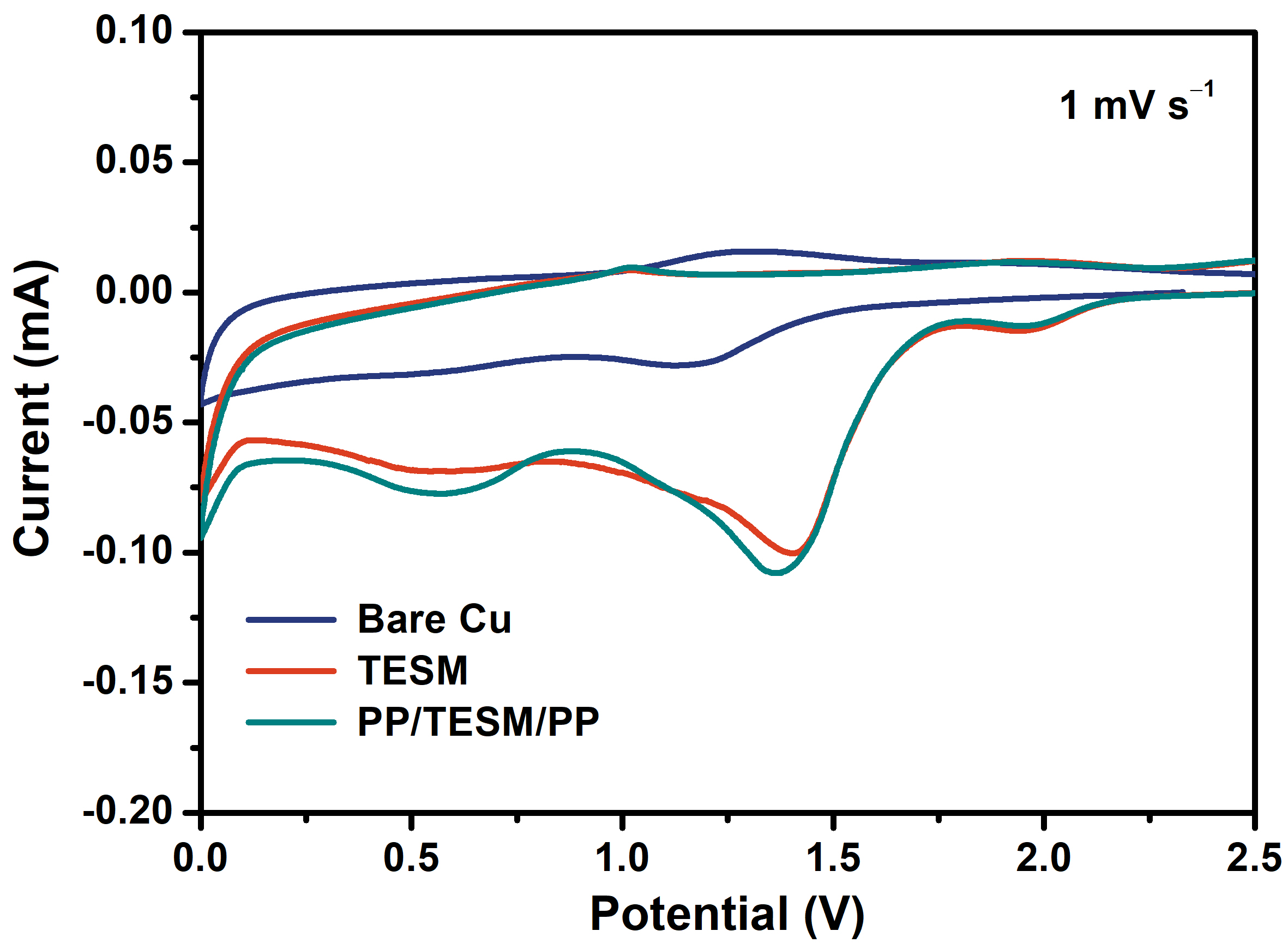


**Supplementary Figure 14.** Cyclic voltammetry of half cells corresponding to bare Cu, TESM, and TESM insulated by double-layer polypropylene (PP) at a scan rate of 1 mV s^-1^ in ether-based electrolytes.


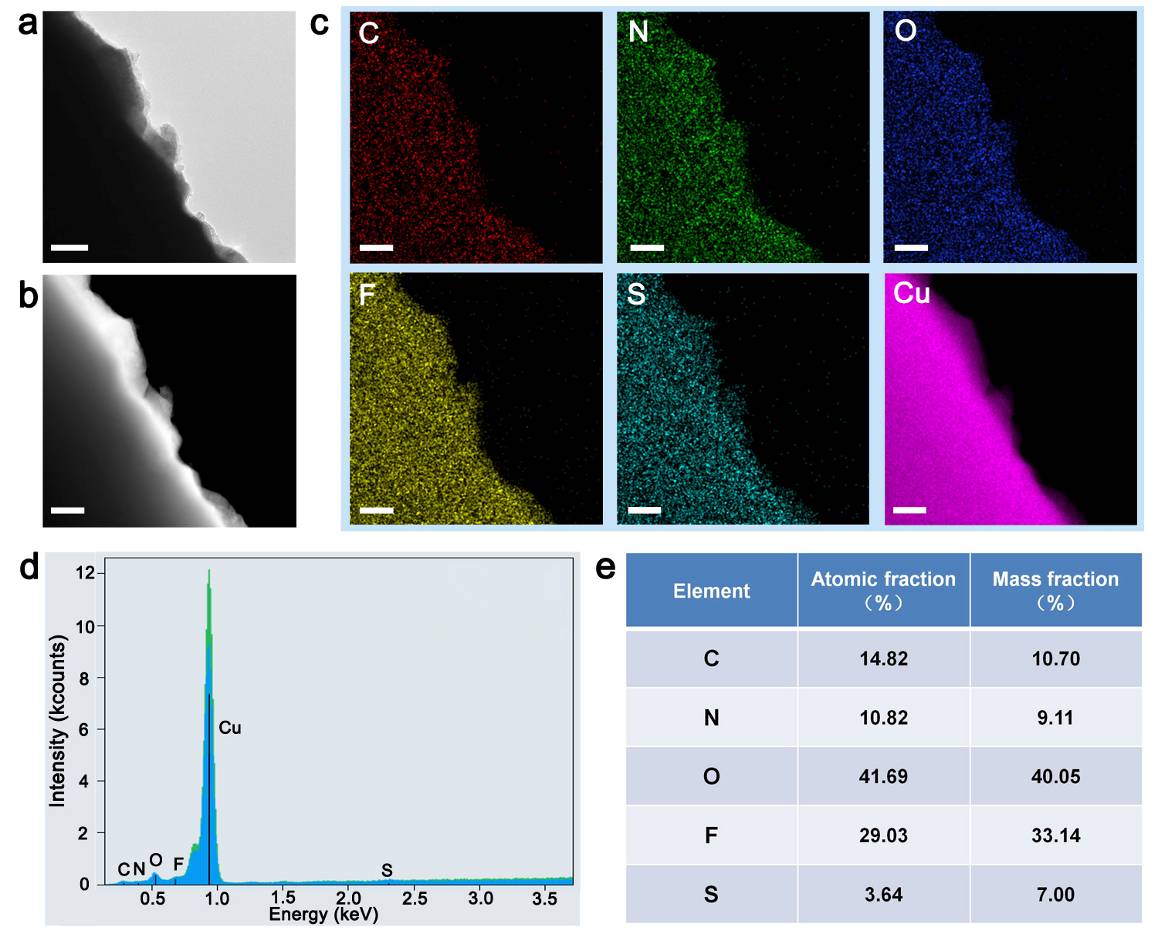


**Supplementary Figure 15.** Charecterization of SEI formed at 1 V without TESM. **a, b** Cryo-TEM and STEM image of the formed SEI at 1 V without TESM in ether-based electrolytes at 0.05 mA cm^−2^. **c** The corresponding elemental mapping images in (**b**). Scale bars, (**a**-**c**) 200 nm. **d** The spectrum of element intensity obtained from (**c**). **e** The relevant atomic and mass fraction of varied elements measured in (**c**).


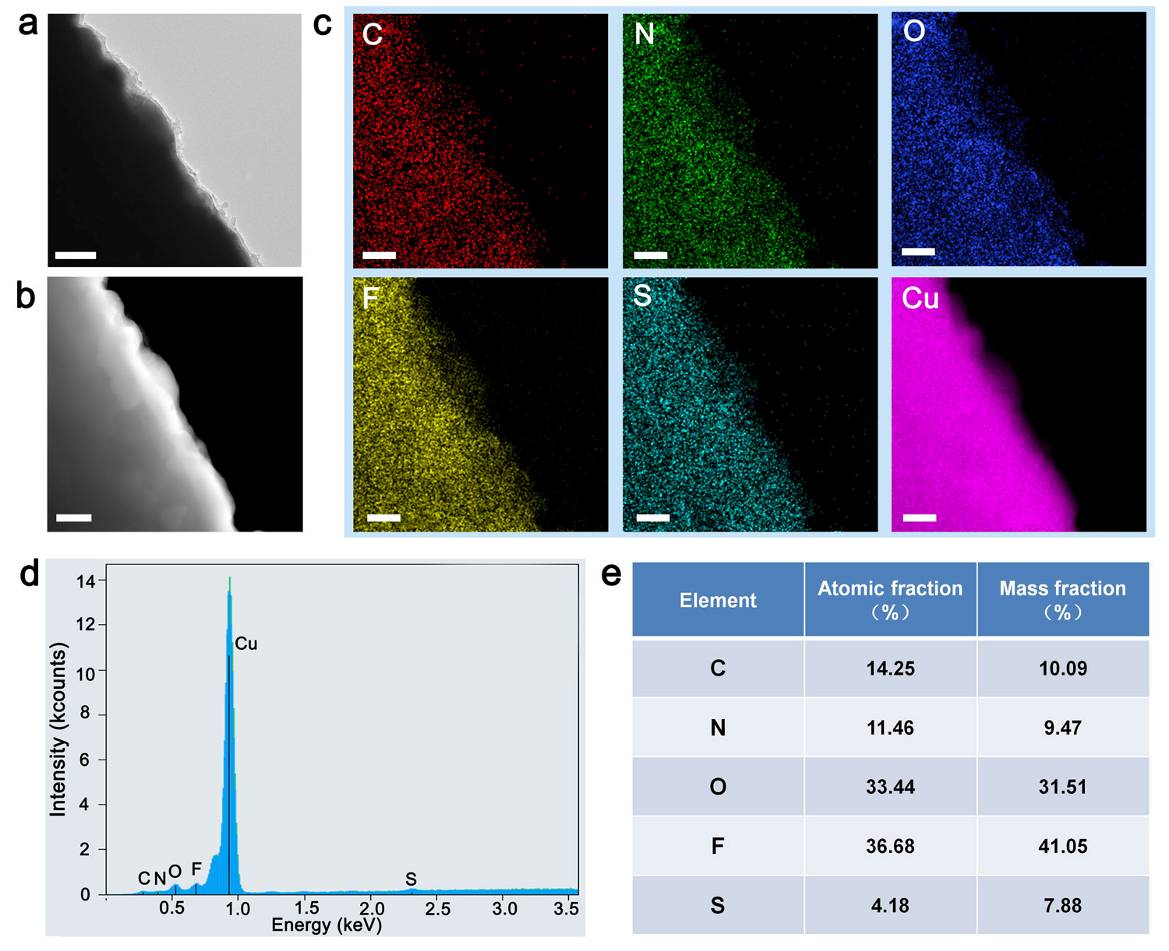


**Supplementary Figure 16.** Charecterization of SEI formed at 1 V with TESM. **a, b** Cryo-TEM and STEM image of the formed SEI at 1 V in the presence of TESM in ether-based electrolytes at 0.05 mA cm^−2^. **c** The corresponding elemental mapping images in (**b**). Scale bars, (**a**-**c**) 200 nm. **d** The spectrum of element intensity obtained from (**c**). **e** The relevant atomic and mass fraction of varied elements measured in (**c**).


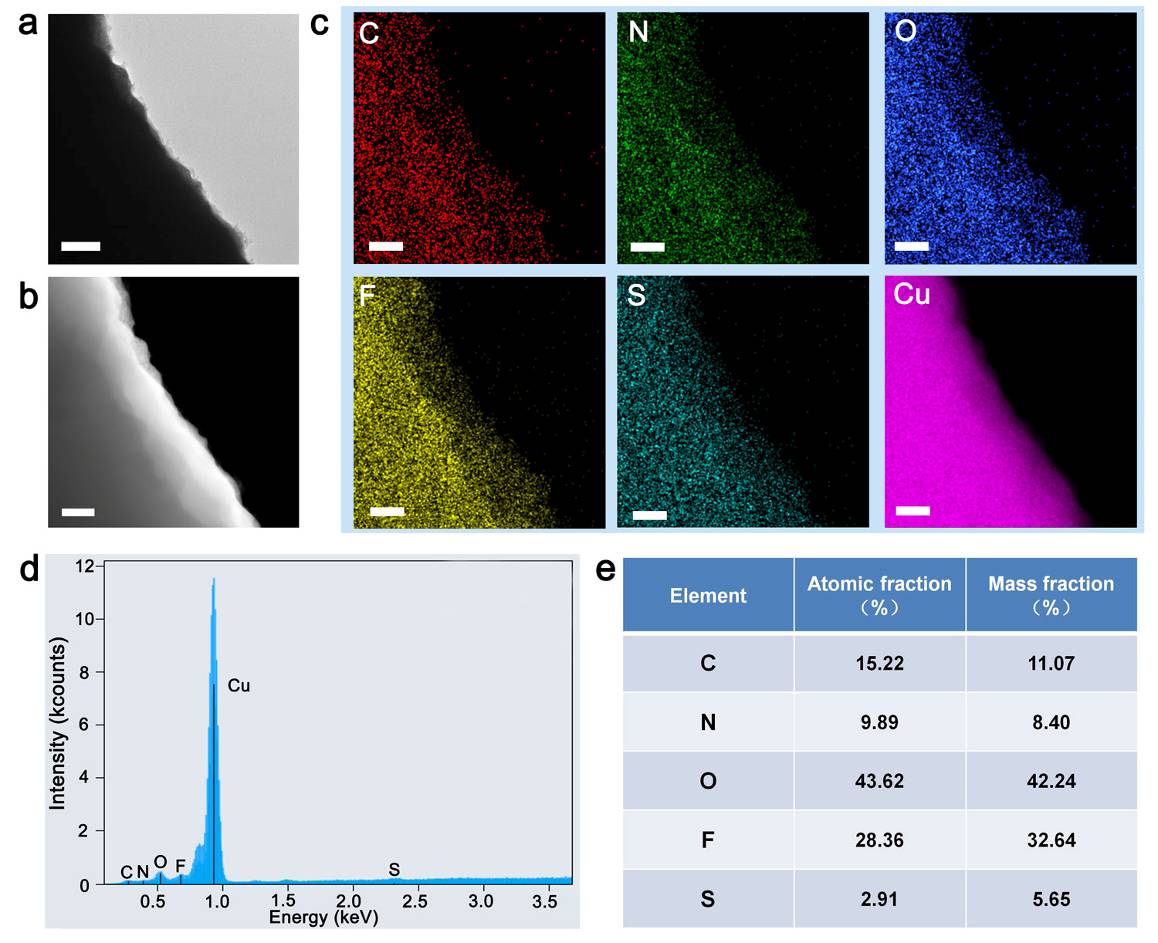


**Supplementary Figure 17.** Charecterization of SEI formed at 0.01 V without TESM. **a, b** Cryo-TEM and STEM image of the formed SEI at 0.01 V without TESM in ether-based electrolytes at 0.05 mA cm^−2^. **c** The corresponding elemental mapping images in (**b**). Scale bars, (**a**-**c**) 200 nm. **d** The spectrum of element intensity obtained from (**c**). **e** The relevant atomic and mass fraction of varied elements measured in (**c**).


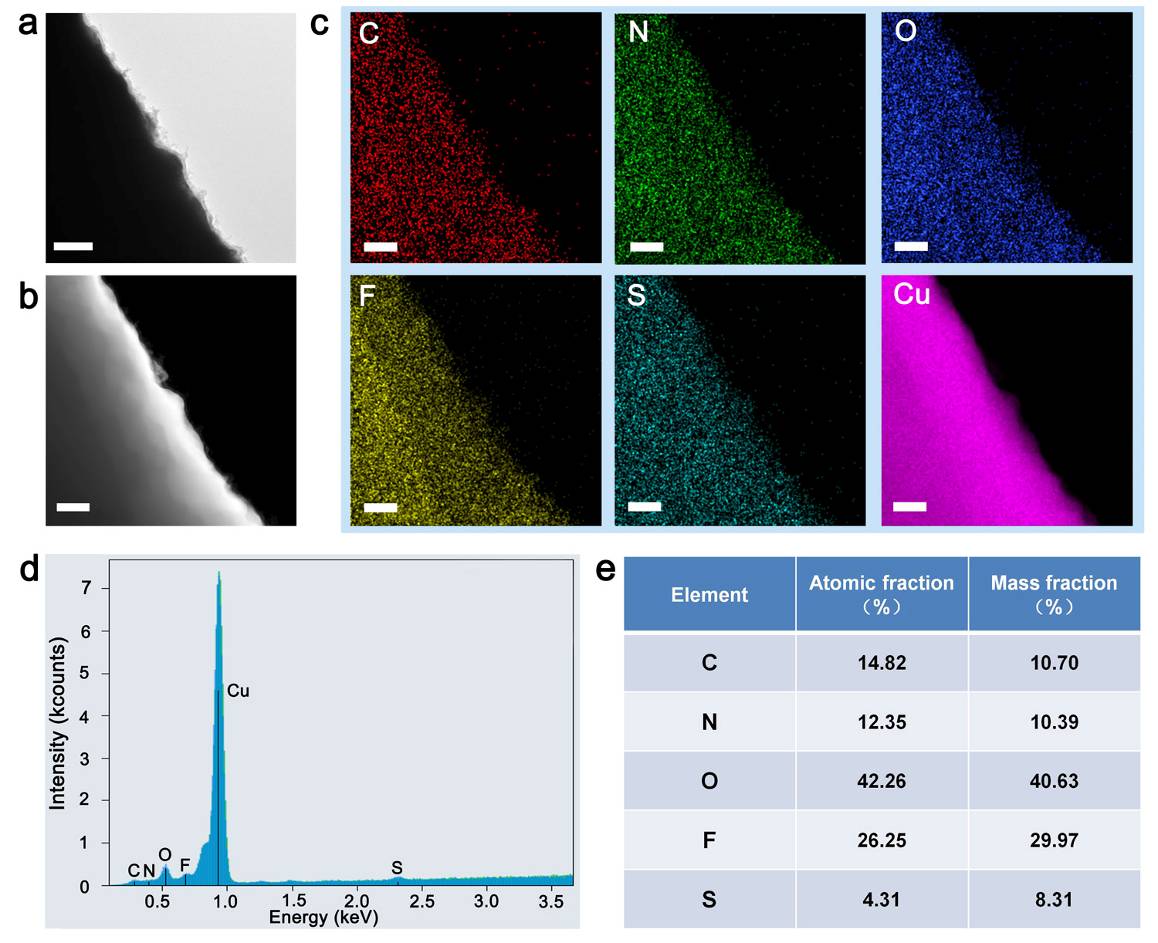


**Supplementary Figure 18.** Charecterization of SEI formed at 0.01 V with TESM. **a, b** Cryo-TEM and STEM image of the formed SEI at 0.01 V in the presence of TESM in ether-based electrolytes at 0.05 mA cm^−2^. **c** The corresponding elemental mapping images in (**b**). Scale bars, (**a**-**c**) 200 nm. **d** The spectrum of element intensity obtained from (**c**). **e** The relevant atomic and mass fraction of varied elements measured in (**c**).


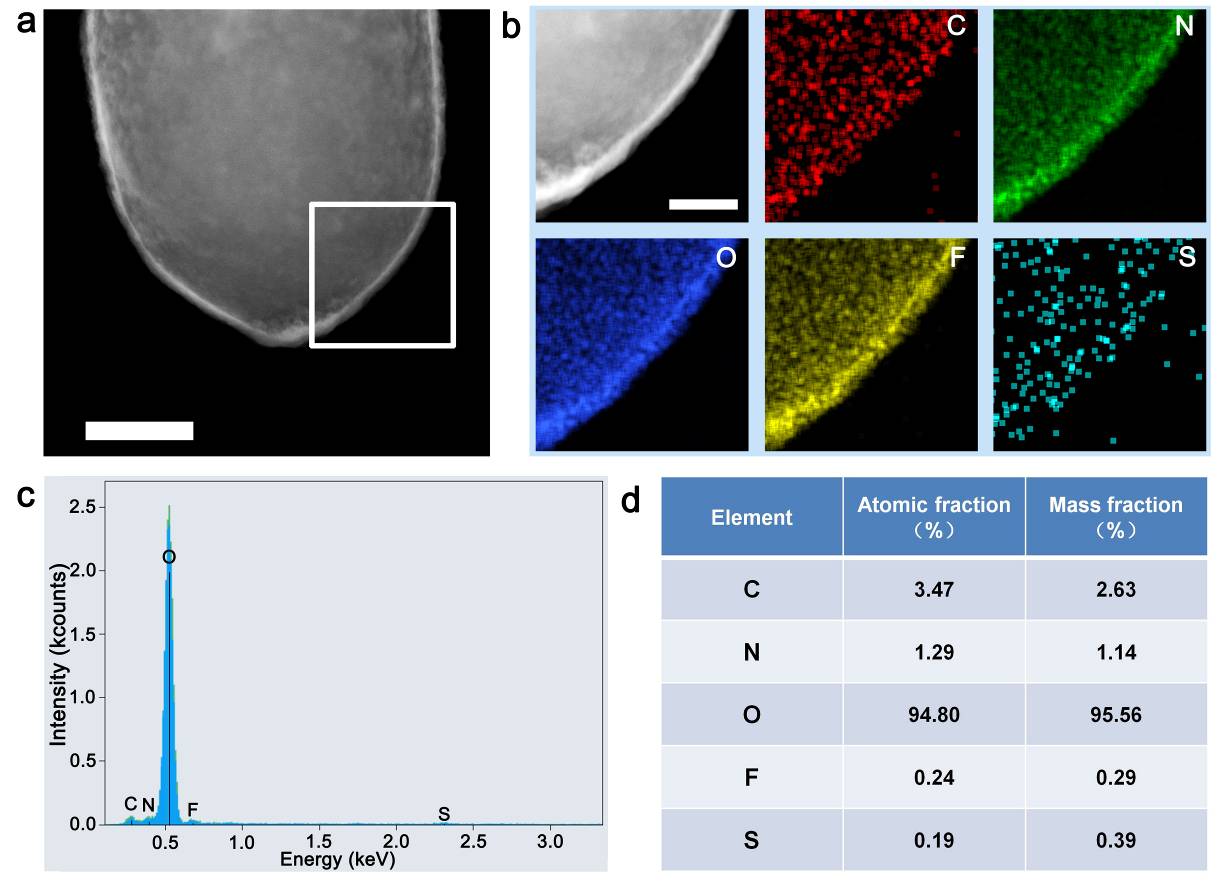


**Supplementary Figure 19.** Charecterization of SEI formed at – 0.05 V without TESM. **a** Cryo-STEM image of the Li deposited at − 0.05 V without TESM in ether-based electrolytes at 1 mA cm^−2^ with a capacity of 0.5 mAh cm^−2^. **b** The corresponding elemental mapping images of the area highlighted in (**a**). Scale bars, (**a**) 500 nm and (**b**) 200 nm. **c** The spectrum of element intensity obtained from (**b**). **d** The relevant atomic and mass fraction of varied elements measured in (**b**).


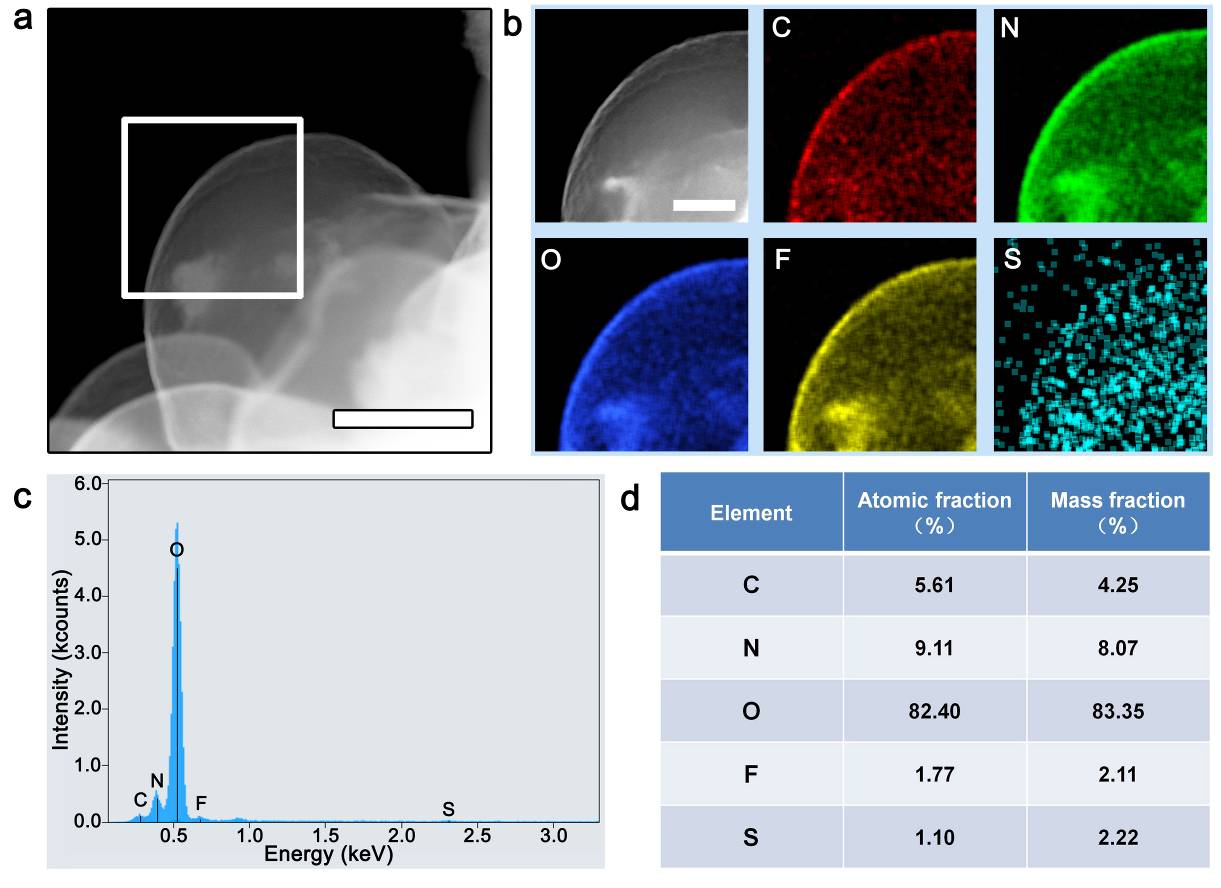


**Supplementary Figure 20.** Charecterization of SEI formed at – 0.05 V with TESM. **a** Cryo-STEM image of the Li sphere formed at **–** 0.05 V in presence of TESM in ether-based electrolytes at 1 mA cm^−2^ with a capacity of 0.5 mAh cm^−2^.**b** The corresponding elemental mapping images of the area highlighted in (**a**). Scale bars, (**a**) 500 nm and (**b**) 200 nm. **c** The spectrum of element intensity obtained from (**b**). **d** The relevant atomic and mass fraction of varied elements measured in (**b**).


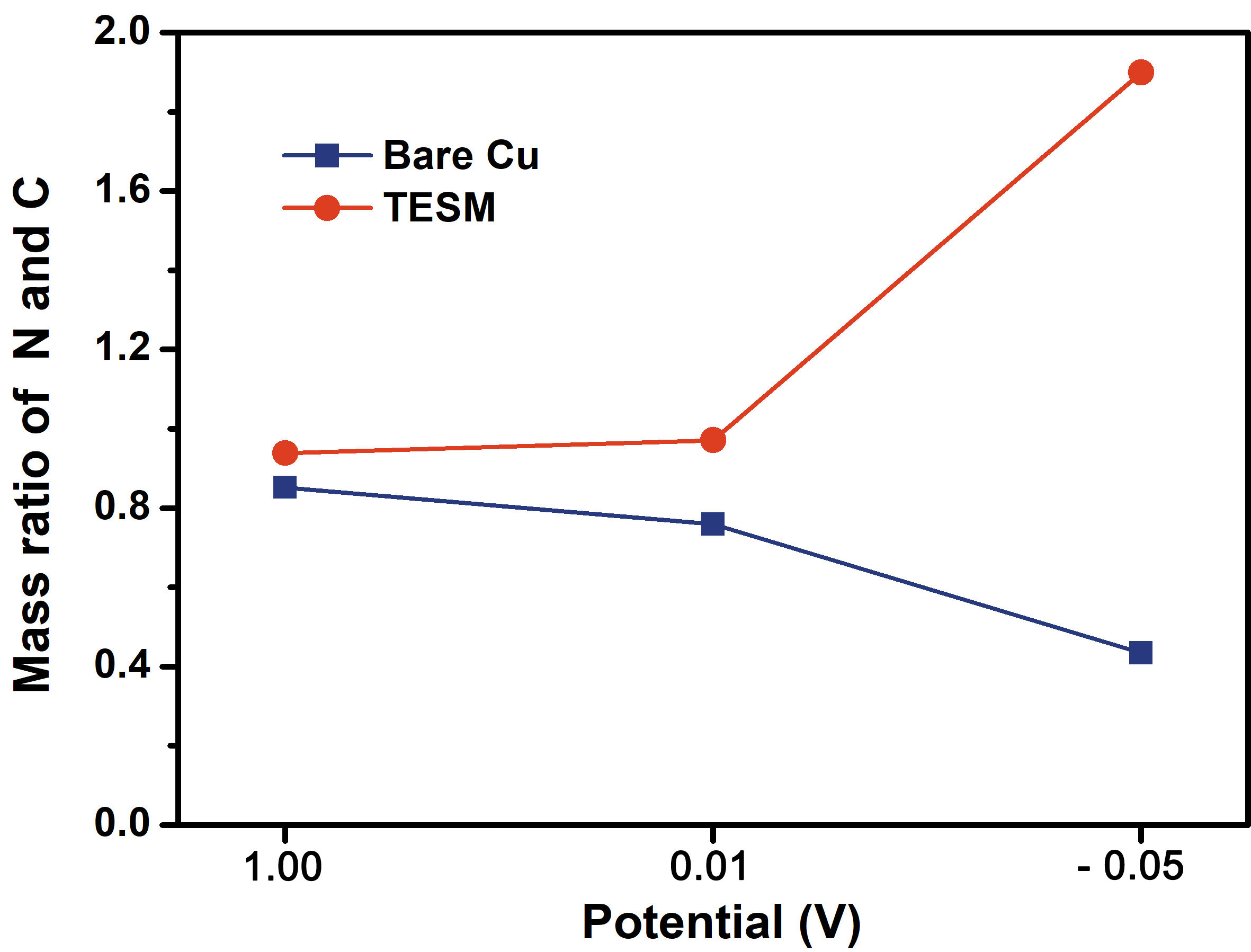


**Supplementary Figure 21.** Mass ratio of N to C at different potentials. The relevant mass ratio of elements N to C within SEI films formed without or with TESM under different potentials in ether-based electrolytes according to Supplementary Fig. 15-20.


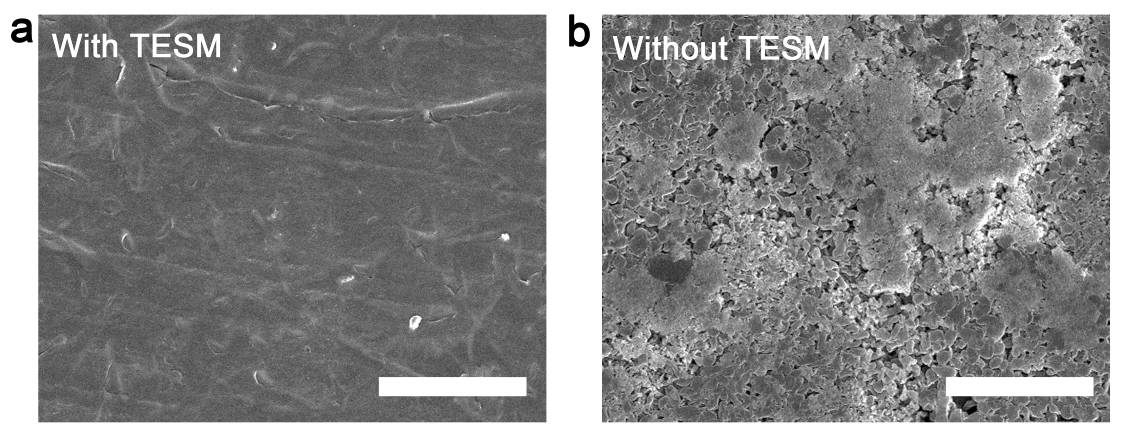


**Supplementary Figure 22.** Morphologies of the cycled Li anodes with and without TESM. **a, b** SEM images of (**a**) the TESM-protected Li anode and (**b**) the unprotected Li anode in ether-based electrolytes at 5 mA cm^−2^ with a Li capacity of 3 mAh cm^−2^ after 20 cycles, respectively. Scale bars, (**a, b)** 50 μm.


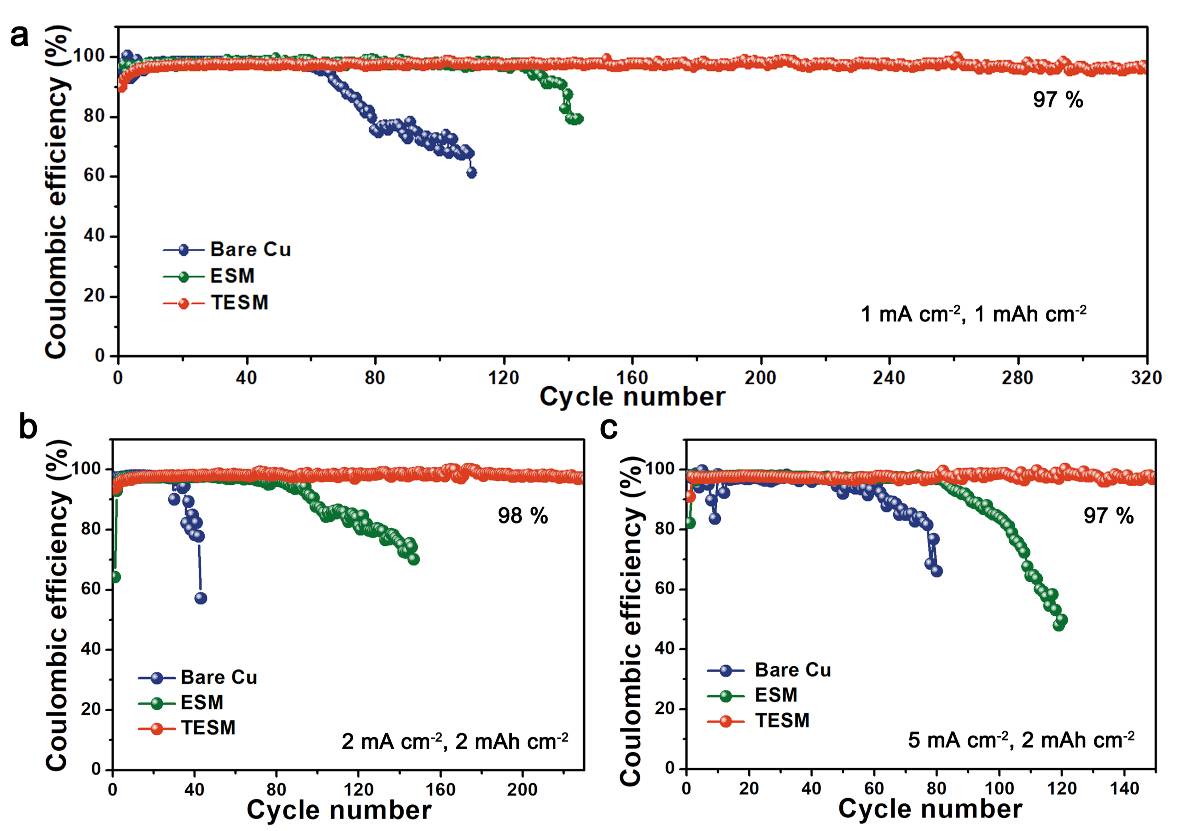


**Supplementary Figure 23.** Coulombic efficiencies of cells with bare Cu, ESM-modified Cu, or TESM-modified Cu in ether-based electrolytes. **a** Long-term electrochemical stability of electrodes at the current density of 1 mA cm^−2^ with a fixed Li capacity of 1 mAh cm^−2^. **b,c** Coulombic efficiencies of electrodes at the current densities of (**b**) 2 mA cm^−2^ and (**c**) 5 mA cm^−2^ with a higher Li capacity of 2 mAh cm^−2^.


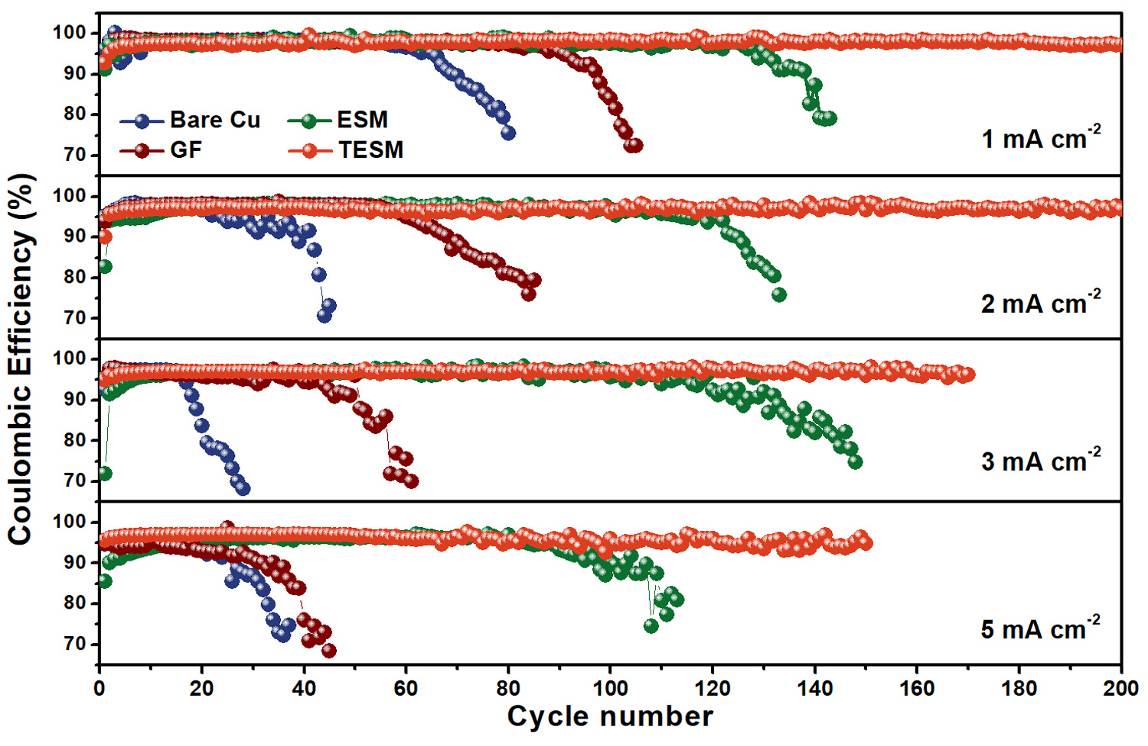


**Supplementary Figure 24.** Comparison of cells in different conditions. Comparison of the Coulombic efficiencies of cells with bare Cu, ESM-modified Cu, TESM-modified Cu, or glass fiber-modified Cu at the current densities of 1, 2, 3 and 5 mA cm^−2^ with a fixed Li capacity of 1 mAh cm^−2^ in ether-based electrolytes.


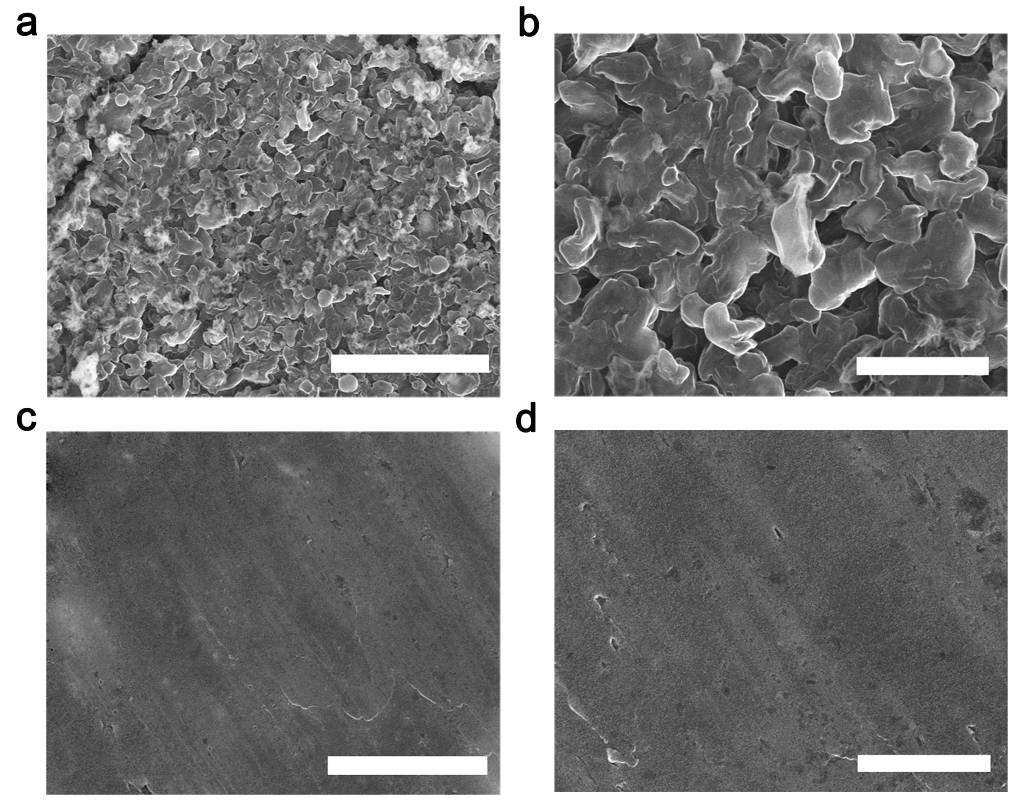


**Supplementary Figure 25.** Morphologies of the Li deposited on Cu current collector upon cycling. **a-d** SEM images of Li deposition on (**a, b**) bare Cu or (**c, d**) TESM modified Cu current collector after 30 cycles at the current density of 2 mA cm^−2^ with fixed Li capacity of 1 mAh cm^−2^ in ether-based electrolytes. Scale bars, (**a, c**) 20 μm, and (**b, d**) 5 μm.


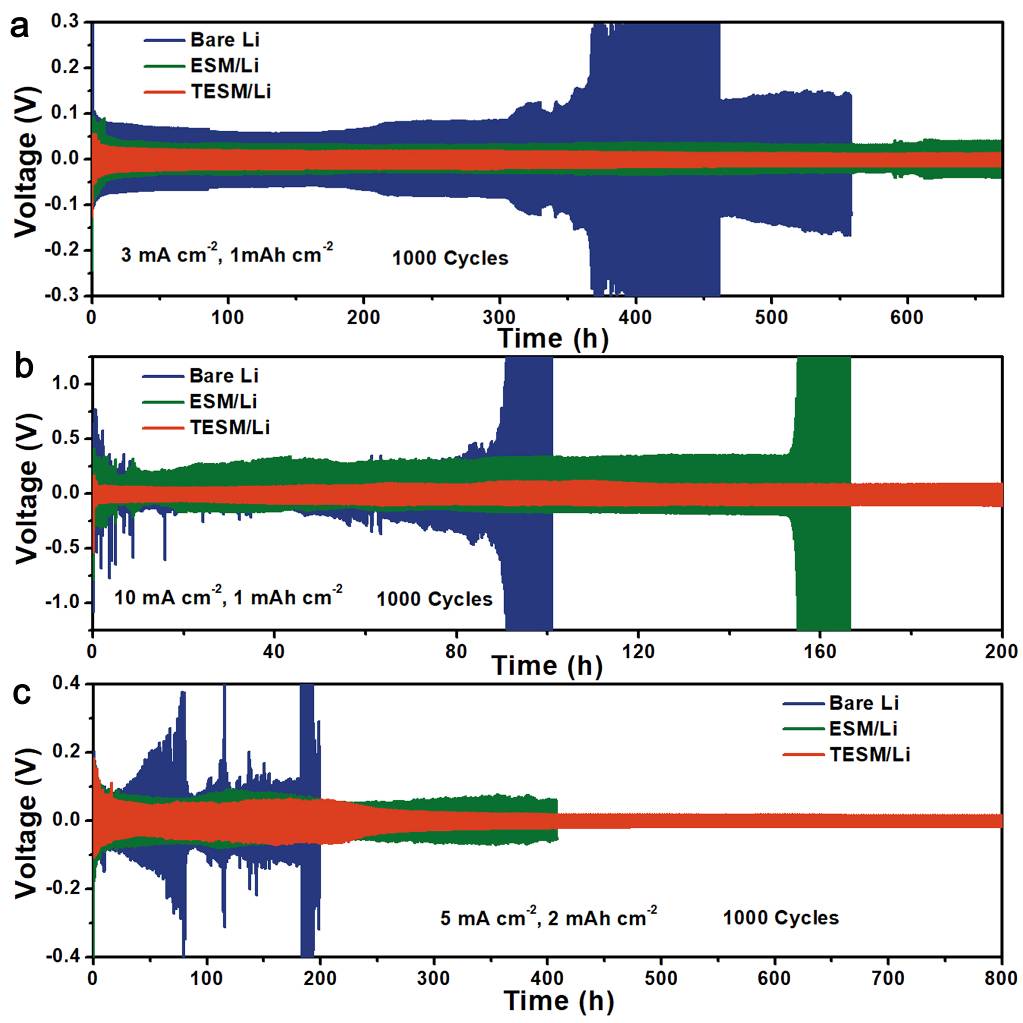


**Supplementary Figure 26.** The performance comparison of cells with different electrodes. **a-c** Comparison of cycling stability performance of electrodes at the current densities of (**a**) 3 mA cm^−2^ and (**b**) 10 mA cm^−2^ with a cycling capacity of 1 mAh cm^−2^ as well as (**c**) 5 mA cm^−2^ with a cycling capacity of 2 mAh cm^−2^ in ether-based electrolytes.


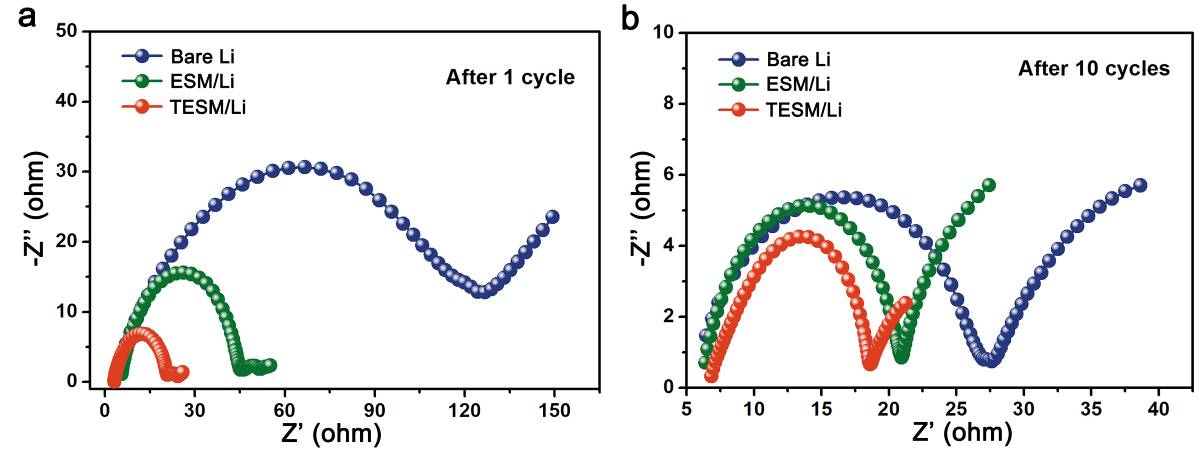


**Supplementary Figure 27.** Comparison of impedance for cells with different electrodes. **a,b** Comparison of impedance of cells with bare Li, ESM/Li, or TESM/Li after (**a**) 1 cycle and (**b**)10 cycles at a current density of 3 mA cm^−2^ with the capacity of 1 mAh cm^−2^ in ether-based electrolytes.

**Supplementary Table 1**. Galvanostatic cycling performance of TESM-modified cells compared with other state-of-the-art modifications.

| Current /Capacity  (mA cm^−2^ / mAh cm^−2^) | Lifespan  (h / cycle) | Overpotential  (mV) | Modifications | Reference |
| --- | --- | --- | --- | --- |
| 1 / 1 | 3000 / 1500  1050 / 525  400 / 200  400 / 200  2000 / 1000 | 15  15  100  40  12 | α-Si3N4 membrane  GZCNT interfacial layer  OIFN film  Li_x_Si coating | 5  6  7  8  This work |
| 3 / 1 | 600 / 900  670 / 1000  133 / 200  133 / 200  670 / 1000 | 25  100  85  150  15 | NGCF host  q-PET host  TiC/C substrate  carbonized wood | 9  10  11  12  This work |
| 5 / 1 | 80 / 200  100 /250  1800 /4500  130 / 325  40 / 100  1200 / 3000 | 50  100  130  160  125  45 | PMF foam  q-PET host  Kimwipe paper  GZCNT interfacial layer  OIFN film | 14  10  13  6  7  This work |
| 10 / 1 | 50 / 250  100 / 500  105 / 525  20 / 100  200 / 1000 | 150  250  500  400  100 | PMF foam  q-PET host  GZCNT interfacial layer  OIFN film | 14  10  6  7  This work |
| 5/2 | 80 / 100  800 / 1000 | 50  18 | q-PET host | 10  This work |

**Supplementary References**

1. Barth A. Infrared spectroscopy of proteins. *Biochim. Biophys. Acta* **1767**, 1073-1101 (2007).

2. Kupser P. et al. Amide-I and -II vibrations of the cyclic β-sheet model peptide gramicidin S in the gas phase. *J. Am. Chem. Soc.* **132**, 2085-2093 (2010).

3. Liang M. et al. Facile in situ synthesis of silver nanoparticles on procyanidin-grafted eggshell membrane and their catalytic properties. *ACS. Appl. Mater. Interfaces* **6**, 4638-4649 (2014).

4. Xiao F. X., Miao J. & Liu B. Layer-by-Layer self-assembly of CdS quantum dots/graphene nanosheets hybrid films for photoelectrochemical and photocatalytic applications. *J. Am. Chem. Soc.* **136**, 1559-1569 (2014).

5. Nan L. et al. Suppressing dendritic lithium formation using porous media in lithium metal based batteries. *Nano Lett.* **18**, 2067-2073 (2018).

6. Zhang H. et al. Lithiophilic-lithiophobic gradient interfacial layer for a highly stable lithium metal anode. *Nat. Commun.* **9**, 3729 (2018).

7. Liu S. et al. In situ solid electrolyte interphase from spray quenching on molten Li: a new way to construct high-performance lithium-metal anodes. *Adv. Mater*. **31,** 1806470 (2018).

8. Tang W. et al. Lithium silicide surface enrichment: a solution to lithium metal battery. *Adv. Mater*. **30,** 1801745 (2018).

9. Liu L. et al. Uniform lithium nucleation/growth induced by lightweight nitrogen-doped graphitic carbon foams for high-performance lithium metal anodes. *Adv. Mater.* **30**, 1706216 (2018).

10. Zhang W., Zhuang H. L., Fan L., Gao L. & Lu Y. A "cation-anion regulation" synergistic anode host for dendrite-free lithium metal batteries. *Sci. Adv.* **4**, eaar4410 (2018).

11. Liu S. et al. 3D TiC/C core/shell nanowire skeleton for dendrite-free and long-life lithium metal anode. *Adv. Energy Mater.* **8**, 1702322 (2018).

12. Zhang Y. et al. High-capacity, low-tortuosity, and channel-guided lithium metal anode. *Proc. Natl. Acad. Sci. USA* **114**, 3584-3589 (2017).

13. Chang C. H., Chung S. H. & Manthiram A. Dendrite-free lithium anode via a homogenous Li-ion distribution enabled by a kimwipe paper. *Adv. Sustain. Syst.* **1**, 1600034 (2017).

14. Fan L. et al. Stable lithium electrodeposition at ultra-high current densities enabled by 3D PMF/Li composite anode. *Adv. Energy Mater.* **8**, 1703360 (2018).
